# Acute kidney injury detection using refined and physiological-feature augmented urine output

**DOI:** 10.1038/s41598-021-97735-0

**Published:** 2021-10-01

**Authors:** Sahar Alkhairy, Leo A. Celi, Mengling Feng, Andrew J. Zimolzak

**Affiliations:** 1 Massachusetts Institute of Technology, Cambridge, MA USA; 2 Beth Israel Deaconess Medical Center, Boston, MA USA; 3 Saw Swee Hock School of Public Health, National University Health System, National University of Singapore, Singapore, Singapore; 4 Baylor College of Medicine, Houston, TX USA; 5 Michael E. DeBakey VA Medical Center, Houston, TX USA

**Keywords:** Computational biology and bioinformatics, Biomarkers, Diseases, Health care, Nephrology, Signs and symptoms, Urology

## Abstract

Acute kidney injury (AKI) is common in the intensive care unit, where it is associated with increased mortality. AKI is often defined using creatinine and urine output criteria. The creatinine-based definition is more reliable but less expedient, whereas the urine output based definition is rapid but less reliable. Our goal is to examine the urine output criterion and augment it with physiological features for better agreement with creatinine-based definitions of AKI. The objectives are threefold: (1) to characterize the baseline agreement of urine output and creatinine definitions of AKI; (2) to refine the urine output criteria to identify the thresholds that best agree with the creatinine-based definition; and (3) to build generalized estimating equation (GEE) and generalized linear mixed-effects (GLME) models with static and time-varying features to improve the accuracy of a near-real-time marker for AKI. We performed a retrospective observational study using data from two independent critical care databases, MIMIC-III and eICU, for critically ill patients who developed AKI in intensive care units. We found that the conventional urine output criterion (6 hr, 0.5 ml/kg/h) has specificity and sensitivity of 0.49 and 0.54 for MIMIC-III database; and specificity and sensitivity of 0.38 and 0.56 for eICU. Secondly, urine output thresholds of 12 hours and 0.6 ml/kg/h have specificity and sensitivity of 0.58 and 0.48 for MIMIC-III; and urine output thresholds of 10 hours and 0.6 ml/kg/h have specificity and sensitivity of 0.49 and 0.48 for eICU. Thirdly, the GEE model of four hours duration augmented with static and time-varying features can achieve a specificity and sensitivity of 0.66 and 0.61 for MIMIC-III; and specificity and sensitivity of 0.66 and 0.64 for eICU. The GLME model of four hours duration augmented with static and time-varying features can achieve a specificity and sensitivity of 0.71 and 0.55 for MIMIC-III; and specificity and sensitivity of 0.66 and 0.60 for eICU. The GEE model has greater performance than the GLME model, however, the GLME model is more reflective of the variables as fixed effects or random effects. The significant improvement in performance, relative to current definitions, when augmenting with patient features, suggest the need of incorporating these features when detecting disease onset and modeling at window-level rather than patient-level.

## Introduction

Acute kidney injury (AKI) is a sudden decrease in kidney function, resulting in fluid dysregulation, electrolyte abnormalities, and/or retention of waste products^1^. Approximately seven percent of patients in hospitals, and over half of patients in intensive care units (ICUs) are thought to develop AKI during hospital stay^2^. Multiple studies have shown a very strong association between AKI and consequent septic shock^3^ and mortality in adults^4–8^ and in children^9^. Early intervention is known to lower the severity of AKI^10^ making rapid prognostication an important goal^11^.

The detection and treatment of AKI, however, can be challenging as the ailment may result from one or more renal insults (pre-renal, post-renal, and/or intrinsic). Existing definitions of AKI (RIFLE, AKIN, and KDIGO) have similar predictive abilities of AKI patients, and have had associated biomarkers of renal injury studied^12,13^.

The RIFLE criteria^14^ stratify AKI risk into five groups: risk, injury, failure, loss, and end stage renal disease. These criteria were validated in studies of tens of thousands of patients^15–17^, and in systematic reviews^18^, all of which correlated the criteria with mortality and/or other adverse outcomes.

The acute kidney injury network (AKIN) criteria^19^ are a modification of RIFLE that have been validated in several studies^20–22^, including one study of over 300,000 patients, thereby making them more popular for research studies^23^. The more recent KDIGO criteria are similar to AKIN in the urine output aspect with more elaborate creatinine aspect^24^.

While the details of the criteria may differ, they are united by their use of creatinine (CR) and urine output (UO) to independently define AKI^25,26^. Furthermore, their lowest level criteria for AKI have a common requirement of a maximum urine output of 0.5 ml/kg/h for at least 6 h and creatinine level of greater than 1.5 $\times$ the baseline.

The independence of the urine output and creatinine definitions, however, often leads to conflicting conclusions. The urine output definition has the advantage of being more readily available (as creatinine is often measured only once a day)^19,27^, but it is also less strongly associated with ICU outcomes than the creatinine definition. This is because the relationship between AKI and urine output depends on the type of renal injury (pre-renal, post-renal, or intrinsic). For example, pre-renal issues are associated with oliguria, post-renal issues often result in anuria, and intrinsic renal issues have varying effects on urine output (sometimes even increasing it), depending on the region injured and the extent of injury.

The relationship between urine output and AKI have been studied in detail^28^. Urine output as a marker of AKI is probably confounded by multiple factors^29^. That is, fluctuations in urine output can be confounded by variables unrelated to AKI. Overall, low urine output may indicate AKI in some patients but not others, and certain clinical variables should be considered before urine output is used to make the diagnosis. Unlike urine output, multiple investigators have indicated a strong preference for the creatinine definition of AKI^2,11^ and have found it to have an overall low false positive rate^30^. However, research has also shown that utilizing both creatinine and UO significantly increase the detection power of AKI as compared to only using creatinine^31,32^.

Because the urine- and creatinine-based definition “limits timely and accurate AKI diagnosis”, a variety of additional biomarkers for AKI have been investigated^33^. The goal is a marker of AKI that is more specific and sensitive than existing criteria, and which ideally becomes detectable before a rise in creatinine. One biomarker clinically available in several countries is neutrophil gelatinase-associated lipocalin (NGAL), and another test, known as “Nephrocheck,” is formed by the combination of two markers of cell cycle arrest^33,34^. Such biomarkers are not measured in all patients, and it is not yet clear when or in what populations they should be measured, as they may add to healthcare costs^29^. Unfortunately, existing biomarkers have shown mixed prognostic ability^35^.

We hypothesize that urine output can indicate AKI before a rise in creatinine, and that improved sensitivity and specificity can be achieved if the time courses of other easily measured physiologic variables are taken into account. This combination could be considered a “digital biomarker,” rather than a chemical one such as NGAL.

Our goals are: (1) to characterize the agreement between the urine output and creatinine definitions for AKI, (2) to determine what time and volume thresholds of the urine definition best agree with the creatinine definition, and (3) build generalized estimating equation (GEE)^36^ and generalized linear mixed-effects (GLME) models^37^ with static and time-varying features to improve agreement with the creatinine-based definition, without sacrificing expediency.

We perform this study on two independent large retrospective clinical archives. We do not intend to formulate a new, unitary definition of AKI that will supplant the measurement of creatinine. Rather, our aim is to determine a urine output-based detector that is more aligned with the creatinine criteria for AKI.

## Methods

### Data set and feature extraction

Data for this study were extracted from two independent intensive care databases with clinical and physiological data, MIMIC-III^38^ and eICU^39^. Multiparameter Intelligent Monitoring in Intensive Care III (MIMIC-III) database includes data from over 38,590 Beth Israel Deaconess Medical Center adult ICU patients. The database covers patients who were admitted between 2008 and 2014 to the adult ICUs at Beth Israel Deaconess Medical Center, a tertiary care university academic medical center located in Boston, Massachusetts. It includes physiologic information from bedside monitors and hospital information systems. The data in MIMIC-III were de-identified, and the use of the database for research was approved by the Institutional Review Boards of the Massachusetts Institute of Technology and Beth Israel Deaconess Medical Center. eICU Collaborative Research Database (eICU), includes patient data from a telehealth system developed by Philips Healthcare. The database includes de-identified clinical and physiological data for more than 139,360 patients admitted to one of 335 units at 208 hospitals between 2014 and 2015.

For each patient sample, we extracted static features including age, gender, first measured weight, height, lean body mass (LBM, derived from the weight, height, and gender), and binary indicators for diabetes, heart disease, cancer, and prior use of diuretics. We also extracted time varying features such as serum creatinine, and hourly measures of urine output, vasopressor use, fluid intake, and mean arterial pressure (MAP) from the first 48 h of ICU stay. These features have been shown to be indicators of AKI^24,25,40–42^. Drugs that were considered vasopressors are: dobutamine, dopamine, epinephrine, isuprel, levophed, vasopressin, milrinone, neosynephrine, norepinephrine, and phenylephrine. We computed fluid balance by subtracting fluid output from input and normalized it by the patient’s first measured weight. Inclusion of features such as diuretics would account for increase in urine output that can be factored out in determining if a patient has AKI.

### Pre-processing and inclusion/exclusion criteria

Patients with less than four hours urine output measurements were excluded. Of those with more than four hourly measures, we excluded any patients with a normalized urine output less than or equal 0.5 ml/kg/h during the first 6 h of admission given that they will require data collected prior to ICU admission which the current databases do not capture. As urine output measures occurred at irregular intervals, we estimated the urine output at the end of the sixth hour, when the measure was not recorded, using interpolation between the two nearest measures. Lastly, we excluded the first urine measurement that inconsistently includes urine output in the Emergency Department, in the operating room or the hospital ward prior to ICU admission.

**Figure 1:**
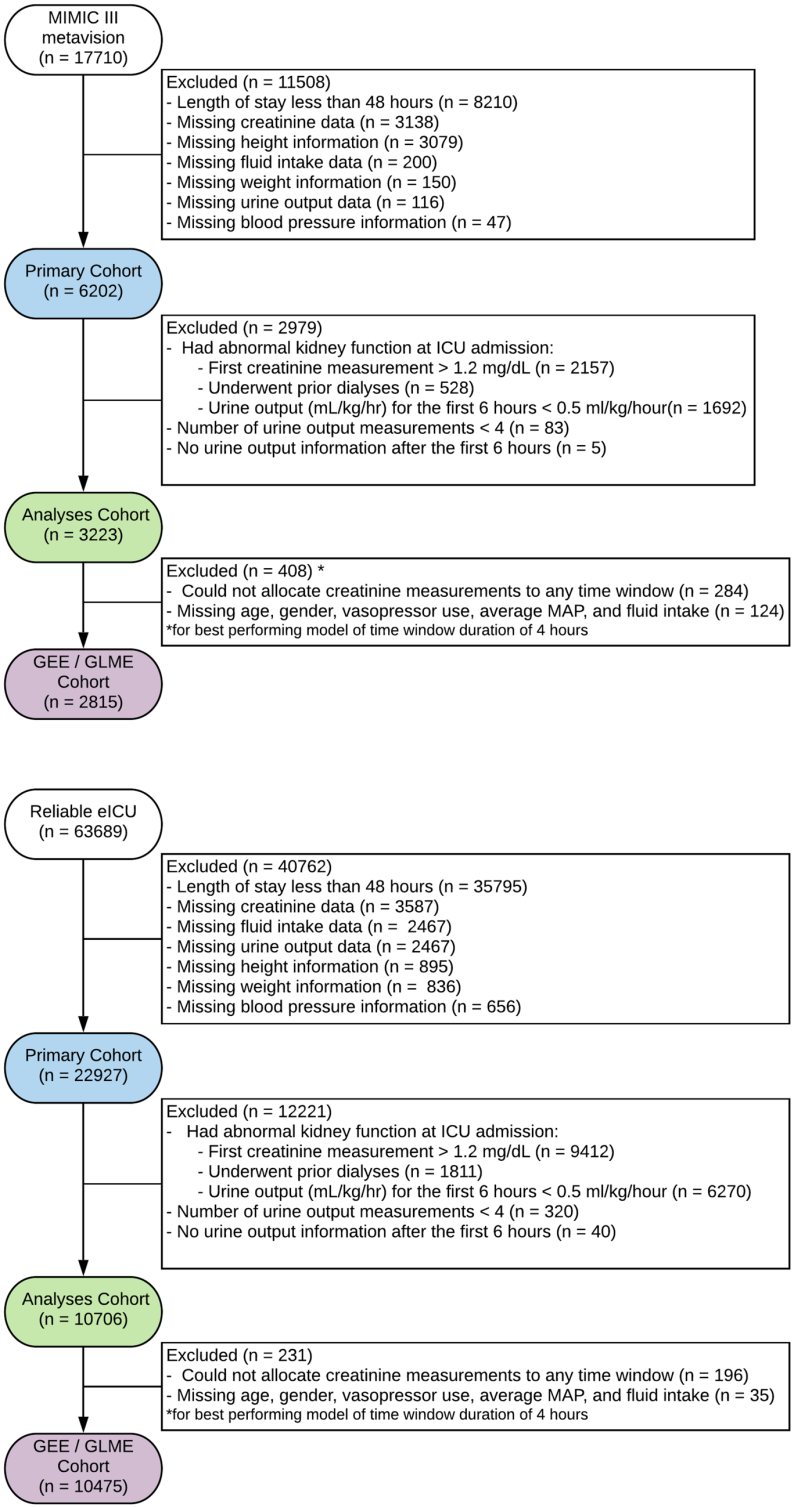
Study schematic of MIMIC-III and eICU cohorts. Study flow-diagram showing initial (primary), non-parametric analysis (analyses), and parametric model GEE (GEE) cohorts of MIMIC-III (top) and eICU (bottom). The initial number of patients in the databases are shown, and detailed exclusion criteria are presented. The numbers of patients who met each criterion does not sum to the total number excluded, as one patient may meet more than one exclusion criterion. *UO* urine output, GEE generalized estimating equation.

We excluded part of the database from analyses because we are concerned only with patients with sufficient data who developed AKI during their ICU stay. The data went through two stages of filtering as illustrated in Fig. 1 . The two cohorts resulting from the two stages are Analyses cohort and subsequently the GEE/GLME cohort.

**Table 1:** Study population characteristics.

Property	MIMIC-III			eICU		
	Primary	Analyses	GEE/GLME	Primary	Analyses	GEE/GLME
Cohort size	6202	3223	2815	22927	10706	10475
Age (years)	67	65.6	65.7	67	64	64
	(54.9, 78.3)	(53.3, 76.9)	(53.3, 76.8)	(55, 77)	(52, 75)	(52, 75)
Gender (male)	3500	1736	1526	12490	5483	5364
	(56.4 %)	(53.9 %)	(54.2 %)	(54.5 %)	(51.2 %)	(51.2 %)
ICU LOS (in days, survivals only)	4.1	5	5.1	3.7	5.1	5.1
	(2.8, 7.3)	(3.1, 8.2)	(3.1, 8.3)	(2.7, 5.9)	(3.3, 8.9)	(3.3, 8.9)
ICU LOS (in days, deceased only)	5.2	3.7	3.6	5	3.5	3.5
	(3.2, 9.4)	(2.5, 6.5)	(2.4, 6.4)	(3.2, 8.7)	(2.6, 5.6)	(2.6, 5.6)
Survival rate	3968	2355	2081	19963	9764	9554
	(64 %)	(73.1 %)	(73.9 %)	(87.1 %)	(91.2 %)	(91.2 %)
First ICU weight(kg)	79.4	75.9	76	80	77.1	77.1
	(66.8, 94.5)	(64.1, 89.3)	(64.5, 89.5)	(66.3, 97.5)	(64, 92.6)	(64, 92.6)
Diabetes	2101	852	755	4866	1696	1668
	(33.9 %)	(26.4 %)	(26.8 %)	(21.2 %)	(15.8 %)	(15.9 %)
Heart disease	4216	2047	1805	2617	846	840
	(68 %)	(63.5 %)	(64.1 %)	(11.4 %)	(7.9 %)	(8 %)
Cancer	1054	549	484	523	342	337
	(17 %)	(17 %)	(17.2 %)	(2.3 %)	(3.2 %)	(3.2 %)
Height (cm)	170	168	168	170	168	168
	(163, 178)	(160, 178)	(160, 178)	(162, 177.8)	(160, 177.8)	(160, 177.8)
First cr	1	0.8	0.8	1.1	0.8	0.8
	(0.8, 1.5)	(0.7, 1)	(0.7, 1)	(0.7, 1.7)	(0.6, 1)	(0.6, 1)
Diuretics	3600	1828	1644	10715	4694	4630
	(58 %)	(56.7 %)	(58.4 %)	(46.7 %)	(43.8 %)	(44.2 %)
LBM	53	51.5	51.7	53.3	51.9	51.9
	(45.6, 60.3)	(44.3, 58.7)	(44.4, 58.7)	(45.9, 60.7)	(44.7, 59.2)	(44.8, 59.2)
Met AKI_Cr definition	4290	1743	1523	12937	3680	3621
	(69.2 %)	(54.1 %)	(54.1 %)	(56.4 %)	(34.4 %)	(34.6 %)

Representation of binary and continuous properties of primary, analyses, and GEE/GLME cohorts. Properties include *LOS* length of stay, *LBM* lean body mass, $AKI_{Cr}$ acute kidney injury based on creatinine. Binary properties are indicated with percentages of positive cases, and continuous properties are indicated with median and interquartile ranges.

**Figure 2:**
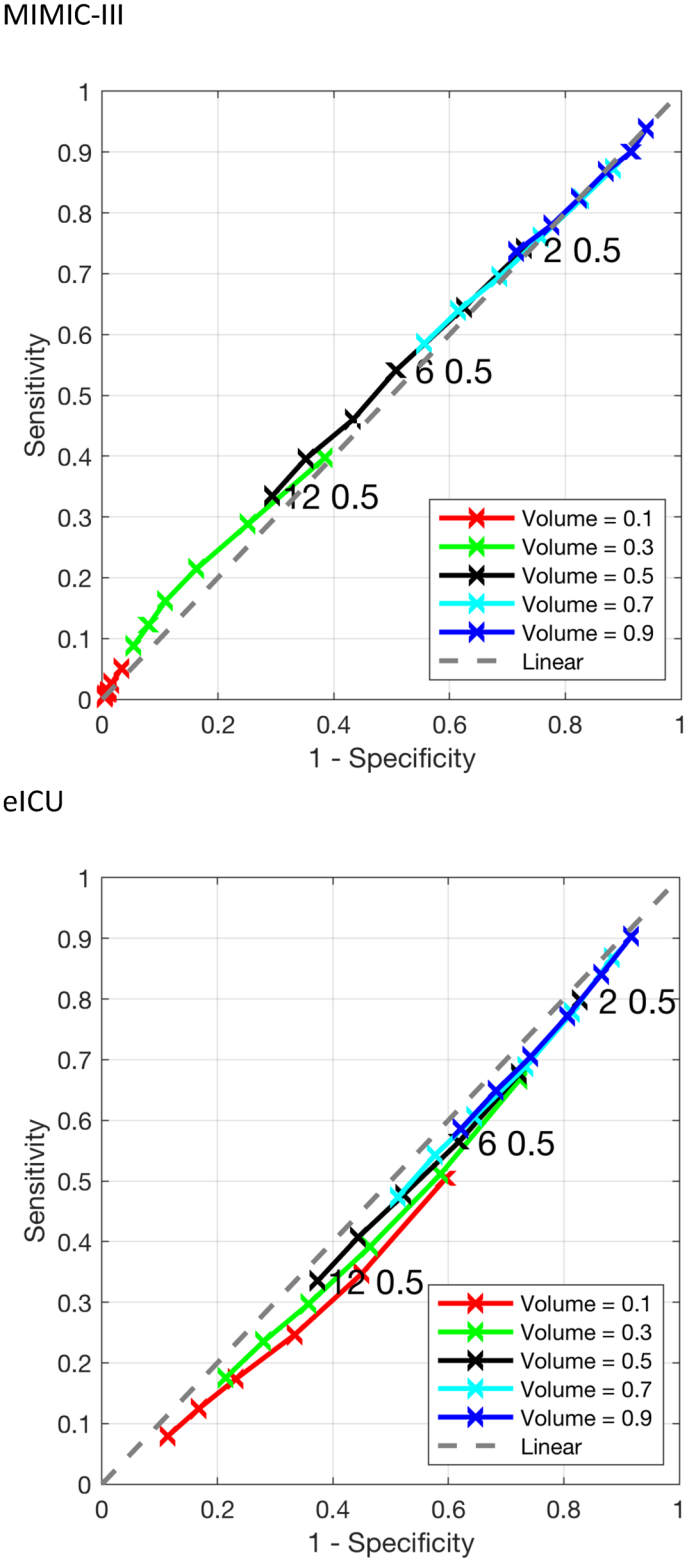
Sensitivity and specificity of various combinations of volume and time thresholds. Performance characteristics of urine output based definition relative to acute kidney injury based on creatinine for MIMIC-III (top) and eICU (bottom). The standard urine output based threshold is shown (labeled with “T $=$ 6, V $=$ 0.5” black line), along with variations on these thresholds. The color of the line corresponds to the volume threshold, while the ticks on each individual line segment represent time thresholds (from 2 to 12 h). Time thresholds increase traveling down and left along a curve. *V* volume (ml/kg/h), *T* time (h). The dashed line represents the ROC curve for a classification that is purely random.

**Figure 3:**
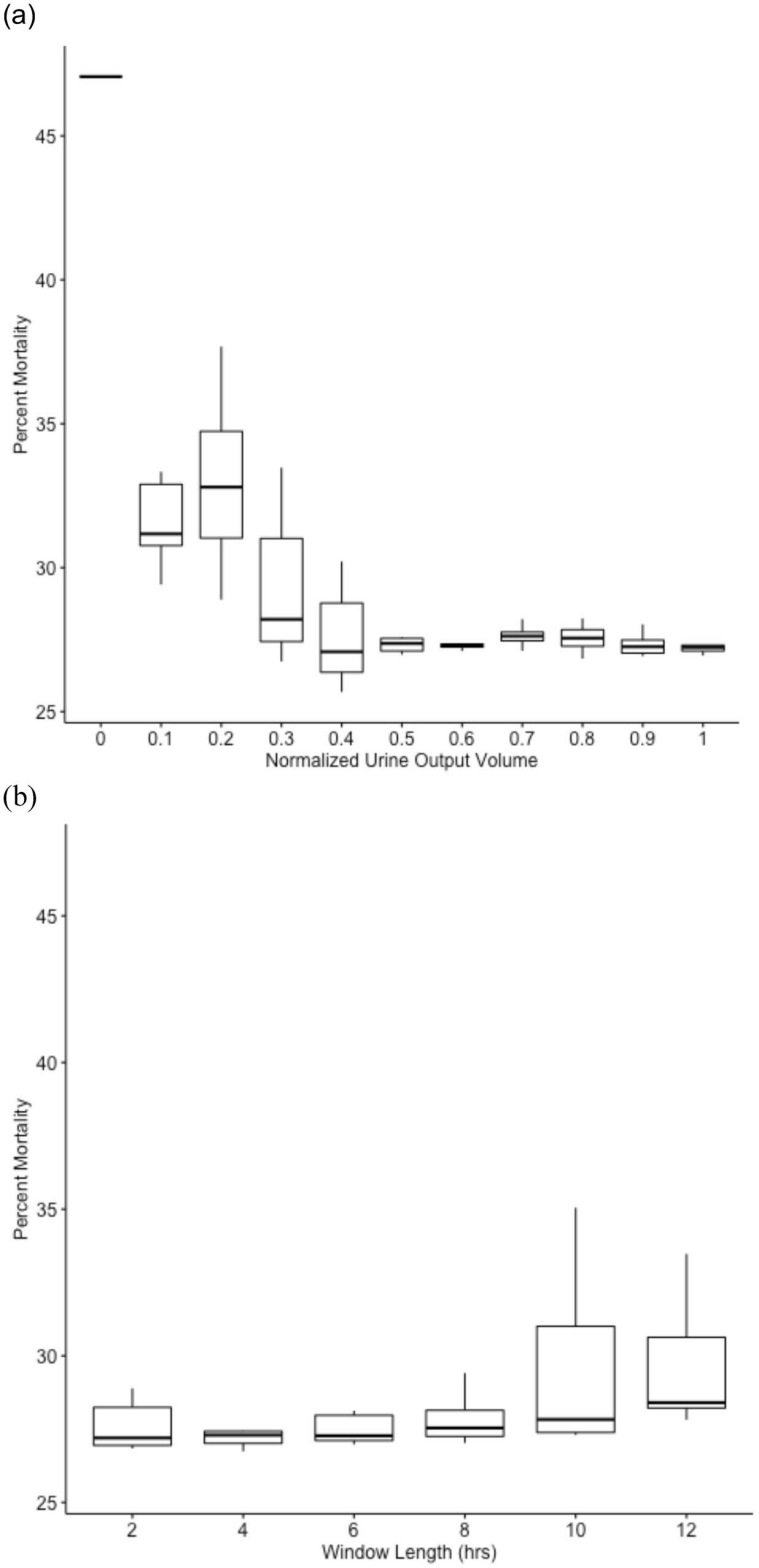
Percent mortality vs normalized urine output and duration thresholds. Boxplots of distribution of percent morality for patients meeting the normalized volume and duration thresholds of urine output-based AKI definition. **(a)** Plots the distribution of percent mortality across the normalized volume thresholds, **(b)** plots the distribution of percent mortality across the urine output window lengths.

**Figure 4:**
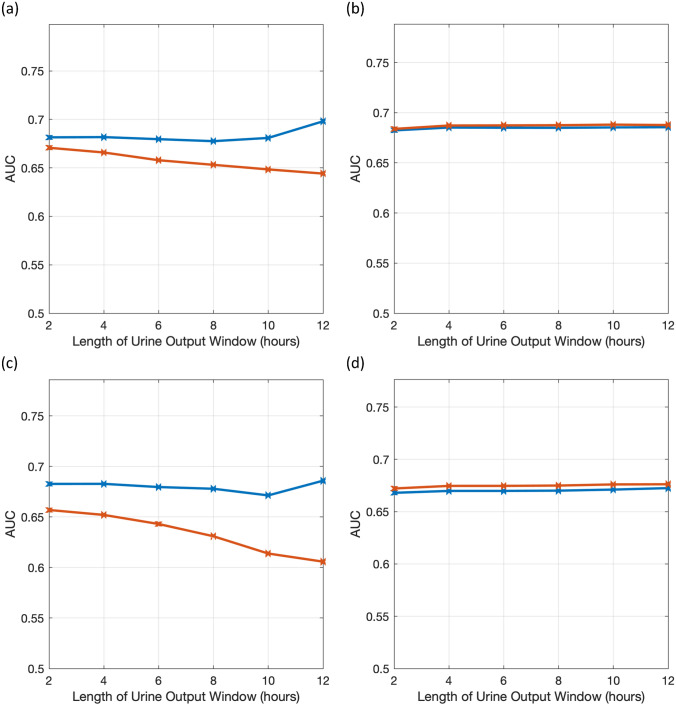
AUC of the GEE and GLME multivariable models. Area under the receiver operating characteristic curve (AUC) are plotted for **(a)** GEE model using MIMIC-III, **(b)** GEE model using eICU, **(c)** GLME model using MIMIC-III, **(d)** GLME model using eICU. Performance of multivariable generalized estimating equation models is plotted against duration of urine output data input to the model. The two curves represent results of training two distinct models on mutually exclusive partitions of the data and using the estimated models on a common test set that does not overlap with the training set. This has been done to confirm consistency of results.

The Analyses cohort is used in characterizing the baseline symmetry between the urine output and creatinine criteria of AKI, and in evaluating the performance of various combinations of time and volume thresholds. It included only patients who had normal kidney function at ICU admission. Therefore, we excluded patients if they had undergone dialyses prior to ICU admission, or if they had a first creatinine measure greater than 1.2 mg/dl, or had an average urine output less than 0.5 ml/kg/h for the first 6 h. Additionally, we excluded patients that had missing data, and ones with too few observations to reliably extract information from (e.g. had less than four measurements of urine output data).

The GEE/GLME cohort is used in identifying a urine output based model that is augmented with other static and dynamic features to predict AKI onset. This cohort is a subset of the Analyses cohort but additionally excluded any patient with missing values for the static and dynamic features used in the model. These features are: age, gender, use of diuretics, use of vasopressors, average MAP, and fluid intake.

### Baseline symmetry and time/volume refinement

All three AKI standards (RIFLE, AKIN, and KDIGO) have similar criteria for their lowest levels of AKI classification. Stage 1 of KDIGO and AKIN and the risk stage of RIFLE require urine output that characterizes AKI by time and volume thresholds of 6 h and 0.5 ml/kg/h and a creatinine level of greater than 1.5 $\times$ the baseline. The creatinine-based criteria for classifying patients as having AKI ($AKI_{Cr}$) is based on the creatinine measurements within the first 48 h of ICU admission where we define AKI as either (1) an increase in creatinine greater than or equal 0.3 mg/dl from hospital stay minimum, or (2) a 50% or more increase from hospital stay minimum^16^. The urine output based criterion ($AKI_{UO}$) classifies patients as having AKI if any time window of a given length threshold has an average weight-normalized urine output less than the volume threshold. We investigated the baseline symmetry between the creatinine and urine criteria of AKI. In particular, we determined its classification performance as indicated by sensitivity and specificity of time and volume thresholds of 6 h and 0.5 ml/kg/h with the creatinine-based definition of AKI as reference. We also refined the choice of time and volume threshold combinations that allowed for the greatest overlap between $AKI_{UO}$ and acute kidney injury based on creatinine ($AKI_{Cr}$). The time thresholds we investigated ranged from 2 to 12 h in increments of 2 while the volume thresholds ranged from 0 to 1 ml/kg/h in increments of 0.1. For each combination of thresholds, we calculated specificity, sensitivity, J-point distance, and net reclassification index (NRI). J-point is the point on the ROC curve that has the least Cartesian distance to 100% sensitivity and specificity.

### Multivariable modeling

Urine output is time-varying, with future values correlated to past values. This makes standard generalized linear modeling approaches invalid. To address this, we employed a generalized estimating equation (GEE), which estimates the parameters of a generalized linear model without any assumptions about the covariance structure of the data, allowing us to use multiple correlated urine observations for model parameter estimation.

The following features were included in the GEE model to predict AKI onset according to the creatinine criteria: age, having diabetes, having heart disease, having cancer, prior diuretic use, prior vasopressor use, first creatinine measure, lean body mass (LBM), time-averaged mean arterial pressure, and fluid balance. All these variables are considered as fixed effects in the GEE model. In comparison, for the GLME model, we consider age, prior vasopressor use, first creatinine measure, LBM, time-averaged mean arterial pressure, and fluid balance to be fixed effects; and a patient having diabetes, heart disease, cancer, and been given diuretic prior as random effects. This better representation could potentially lead to greater agreement with creatinine-based definition. The GLME model integrates out the random effects, but is limited to categorical variables. The extended GLMM model^43^ is able to model continuous random effects using Monte Carlo simulation and expectation maximization, which makes it computationally infeasible for the size of the database we are using.

We computed fluid balance within a certain time window by subtracting the total urine output within the window from the adjusted fluid intake and normalizing it by the patient’s first measured weight. The adjusted fluid intake is the sum of fluid intake up to and including during the time window minus the total urine output up to the start of the time window.

As in our refinement analyses, we explored various time window lengths and observed their impact on model performance in prediction of AKI onset with reference to creatinine based AKI criteria. Specifically, we explored time thresholds ranging from 2 to 12 h in increments of 2.

We generated the GEE model using GEEQBOX toolkit^36^ and the GLME model using Matlab’s GLME function using a randomly selected training set comprising of two-thirds of the GEE/GLME cohort, and tested the performance of our fitted models by predicting $AKI_{Cr}$ on the unseen test set (one-third of GEE/GLME cohort). We plotted the receiver operating characteristic (ROC) curve for each of the six models (one model for each time window), and examined the model coefficients, odds ratios, 95% confidence intervals, and p-values for each model.

For each model, we calculated the area under the ROC curve (AUC), J-point specificity and sensitivity, J-point distance, and net reclassification index (NRI). For computing the NRI for the various models, we binarized the prediction of AKI for the validation set using the probability threshold of the J-point.

### Model variables

In order to obtain the features, we extracted the average UO per window per time threshold the same way we computed $AKI_{UO}$.

For the other time-varying features (1) MAP (2) fluid balance (3) use of vasopressors, we used the normalized start time of each window. For the MAP, we obtained the median value one to three hours prior. For the fluid balance, we obtained the difference between fluid input and output and normalized it by weight. For the vasopressors, we checked to see if any vasopressor was used prior to the start time of the window.

To obtain $AKI_{Cr}$ for each window, we labeled each creatinine measurement with 0 or 1 (0: no AKI, 1: has AKI) based on the $AKI_{Cr}$ definition. We also, removed any UO window that overlap with serum creatinine measurements (because it is difficult to know which measurement it would belong to) and any window after the last measurement. We labeled each window based on the next nearest creatinine measurement.

### Net reclassification index

In order to measure the improvement in performance of the various refinements in time and volume thresholds and GEE/GLME models with respect to the standard urine output threshold of 0.5 ml/kg/h for a duration of at least 6 h, we computed their net reclassification improvement (NRI)^44,45^. NRI is the difference between the probability of correct reclassification and the probability of incorrect reclassification. It is also the difference between the sum of the sensitivity and specificity of the new model and the sum of the sensitivity and specificity of the old model.

### Use of experimental animals, and human participants

This is a retrospective study using openly available datasets and does not deal with human participants or groups. Therefore, need for consent is not applicable. Only computational methods were used and no clinical or experimental methods were carried out. All methods were carried out in accordance with relevant guidelines and regulations.

## Results

Characteristics of patients and population sizes for the Primary cohort, Analyses cohort, and cohort of best performing GEE/GLME model for the MIMIC-III and eICU databases are shown in Table 1. We note that the GEE/GLME cohort differs from the Primary cohort in all characteristics in both databases with the exception of cancer indicator, use of diuretics, height, and age in MIMIC-III; and age in eICU . This is to be expected as we only include patients with specific characteristics from the general and heterogeneous patient population.

We also note a significant difference in the number of patients that have heart disease and that have cancer between the MIMIC and eICU databases—heart disease (MIMIC: 68%, eICU: 11.4%), cancer (MIMIC: 17%, eICU: 2.3%). The diagnoses included in the heart disease and cancer categories for MIMIC and eICU include similar diverse set of diagnoses. Johnson et al.^38^ had similar statistics for the percentage of patients with heart disease (71.4%) and Pollard et al.^39^ mentioned that 11.15% and 4.7% of the patients in the eICU had heart disease and cancer respectively, similar to our findings. Supported by existing work, the differences in the percentages of patients with diseases between the MIMIC and eICU datasets suggest that the two sets of patients are significantly different.

**Table 2:** GEE and GLME multivariable models’ estimated parameters.

Variable	OR	95% CI (lower)	95% CI (upper)
**GEE MIMIC-III**			
First creatinine (mg/dL)	5.53227985	3.18738235	9.60131434
Heart disease	0.68956108	0.53381817	0.89065334
Lean body mass (kg)	0.97892524	0.96666817	0.99133774
Prior vasopressors use	0.68976798	0.54471095	0.87345384
Fluid balance (mL/kg)	1.00320513	1.00000000	1.00642052
**GEE eICU**			
Diuretics	1.73100125	1.54187622	1.94332418
First creatinine (mg/dL)	10.66108291	7.91294479	14.36363981
Lean body mass (Kg)	0.979414823	0.97404283	0.98481645
Prior vasopressors use	1.79122546	1.435624578	2.23513136
Prior MAP	0.99481350	0.99183353	0.99780242
Fluid balance (mL/Kg)	1.00200200	1.00140098	1.00250313
**GLME MIMIC-III**			
First creatinine (mg/dL)	43.84672370	15.91878725	120.77146008
Lean body mass (Kg)	0.958773898	0.93828644	0.97961073
Prior vasopressors use	0.46342997	0.30608269	0.70159427
Fluid balance (mL/Kg)	1.00772972	1.00200200	1.01349018
**GLME eICU**			
First creatinine (mg/dL)	483.3301690828	254.49978724	918.00239541
Lean body mass (Kg)	0.95027867	0.93847412	0.96223170
Prior vasopressors use	3.47536670	2.17363308	5.55667551
Prior MAP	0.99292515	0.98738030	0.99860098
Fluid balance (mL/Kg)	1.00320513	1.00220242	1.00430926

Estimated parameters of best performing GEE and GLME models based on AUC values, which has a 4 h window length: odds ratio, and 95% confidence interval for the significant covariates for MIMIC-III and eICU.

**Table 3:** Performance metrics across various models.

Model	Time duration	Sensitivity	Specificity	J-point distance	NRI
**MIMIC**					
Standard urine-based AKI def.	6	0.54102	0.49257	0.68421	–
Non-parametric model— smallest distance	12	0.483	0.57763	0.6676	0.026837
Parametric GEE model—6 h	6	0.65113	0.6186	0.51688	0.21054
Parametric GEE model—greatest AUC	4	0.61437	0.66253	0.51244	0.25559
Parametric GLME model—6 h	6	0.5653	0.65465	0.55519	0.186
Parametric GLME model—greatest AUC	4	0.55017	0.70726	0.5367	0.25192
**eICU**					
Standard urine-based AKI def.	6	0.56413	0.38201	0.75624	-
Non-parametric model— smallest distance	10	0.48354	0.48946	0.72621	0.026444
Parametric GEE model—6 h	6	0.65216	0.64352	0.49807	0.34082
Parametric GEE model—greatest AUC	4	0.63978	0.65514	0.49868	0.34973
Parametric GLME model—6 h	6	0.60618	0.65865	0.52117	0.31366
Parametric GLME model—greatest AUC	4	0.60488	0.65968	0.52147	0.31117

Performance metrics for the standard urine-based AKI definition, best performing non-parametric model, GEE model with six hours duration, best performing GEE model, GLME model with six hours duration, and best performing GLME model. Top table is for the MIMIC-III dataset and the bottom is for eICU.

Additionally, there was a noticeable drop in the percentage of patients that meet the creatinine-based definition of AKI in the eICU database between the Primary and Analyses cohorts (56.4–34.4%). The reason behind this drop is due to there being a large intersection between the patients with abnormal kidney function at ICU admission and the ones who meet the definition of developing creatinine-based AKI. When filtering out the ones with prior abnormal kidney function from the Primary cohort a significant portion of the patients that had further increase in creatinine during their ICU stay were also excluded resulting in the sharp decrease.

The congruence between creatinine-based definition of AKI and mortality has a sensitivity of 0.61 and specificity of 0.48 for MIMIC-III; and sensitivity of 0.47 and specificity of 0.67 for eICU. The baseline symmetry between the standard AKI ($AKI_{UO}$) definition of urine output less than 0.5 ml/kg/h for 6 h and the reference AKI ($AKI_{Cr}$) definition based on creatinine levels has a sensitivity of 0.54 and specificity of 0.49, with a distance of 0.68 from 100% sensitivity and specificity for the MIMIC-III database; and a sensitivity of 0.56 and specificity of 0.38, with a distance of 0.76 from 100% sensitivity and specificity for the eICU database.

The results of refining AKI urine output and time thresholds are depicted in Fig. 2 and supplementary Table S1. For each of the two databases MIMIC-III and eICU, there are volume and time threshold combinations for the urine-based AKI definition that have better congruence with the creatinine-based AKI definition than the standard volume and time thresholds of 0.5 ml/kg/h and 6 h.

For the MIMIC-III database, ranking based on J-point distance results in the optimal time and volume thresholds of $AKI_{UO}$ as UO less than 0.6 ml/kg/h for 12 h. This combination has a sensitivity of 0.48, specificity of 0.58, J-point distance of 0.67, and NRI of 0.027. Ranking the threshold combinations based on NRI values, results in the same optimal time and volume thresholds of $AKI_{UO}$. For the eICU database, ranking based on J-point distance results in the optimal time and volume thresholds of $AKI_{UO}$ as UO less than 0.6 ml/kg/h for 10 h. This combination has a sensitivity of 0.48, specificity of 0.49, distance of 0.73 from 100% sensitivity and specificity, and NRI of 0.026. Ranking the threshold combinations based on NRI values, results in the optimal time and volume thresholds of $AKI_{UO}$ as UO less than 1 ml/kg/h for 2 h. This combination has a sensitivity of 0.92, specificity of 0.074, distance of 0.93 from 100% sensitivity and specificity, and NRI of 0.046.

The mortality percentage of patients meeting the volume and duration thresholds of urine-based definition of AKI decreases as the normalized urine output threshold increases and increases as the time duration threshold increases as shown in Fig. 3.

The area under the ROC curve (AUC) for the GEE/GLME multivariable models augmented physiological features for two partitions are plotted in Fig. 4.

Performance trend across partitions is generally consistent. Ranking of each of GEE and GLME models according to AUC values results in a best performing model with a time window of 4 h for both MIMIC-III and eICU.

The GEE model with a time window of 6 h—the same duration of data as the standard criteria– has a sensitivity of 0.65, a specificity of 0.62, J-point distance of 0.517, and NRI of 0.21 for MIMIC-III; and sensitivity of 0.65, a specificity of 0.64, J-point distance of 0.50, and NRI of 0.34 for eICU. The GLME model with a time window of 6 h has a sensitivity of 0.57, a specificity of 0.65, J-point distance of 0.56 and NRI of 0.19 for MIMIC-III; and sensitivity of 0.61, a specificity of 0.66, J-point distance of 0.52, and NRI of 0.31 for eICU.

The best performing GEE model has a sensitivity of 0.61, specificity of 0.66, J-point distance of 0.512, and NRI of 0.256 for MIMIC-III; and a sensitivity of 0.64, specificity of 0.66, J-point distance of 0.50, and NRI of 0.35 for eICU. The best performing GLME model has a sensitivity of 0.55, specificity of 0.71, J-point distance of 0.54, and NRI of 0.25, for MIMIC-III; and a sensitivity of 0.60, specificity of 0.66, J-point distance of 0.52, and NRI of 0.31, for eICU.

GEE model has better performance than the GLME model for MIMIC and eICU databases. However, we include the GLME model as it is more reflective of fixed and random effects, integrating out random effects.

For the best performing model according to AUC (4 h of data), the odds ratio, and 95% confidence intervals for significant features are tabulated in Table 2.

First creatinine measurement, LBM, prior vasopressor use, and fluid balance were found to exhibit a statistically significant association with $AKI_{Cr}$ in both MIMIC-III and eICU. Additionally, heart disease was a significant indicator in MIMIC-III in the GEE model, while diuretics use and MAP were significant features in eICU in the GEE model. Specifically, increased first creatinine measurement, positive fluid balance, and decreased LBM showed a positive association with AKI. In MIMIC-III, heart disease and vasopressor use showed negative association with AKI. In eICU, use of diuretics and vasopressors use showed positive association, whereas mean arterial pressure showed negative association.

Summary of performance across the various non-parametric and parametric models is tabulated in Table 3. For both databases, MIMIC-III and eICU, J-point distance is reduced for non-parametric model over the standard urine-based AKI definition. Additionally, the distance is substantially reduced for the parametric GEE and GLME models over the non-parametric model.

We also tested the MIMIC-trained model on eICU and vice versa using both GEE and GLME models. The significantly lower performance compared to models trained and tested on the same database leads to the conclusion that there are significant differences between the patients cohorts not captured in the databases. These differences may partially arise from distinctions in qualitative procedures and quantitative variables not part of the database.

## Discussion

Over the past 3 decades, the incidence of AKI has increased over 20-fold, making it an important problem in critical care medicine. The purpose of this paper was to investigate the complex factors mediating the relationship between urine output and creatinine in AKI, and to develop a time varying multivariable model that identifies factors mediating the relationship based on augmentation of urine output with physiological features.

For the diagnosis of AKI, serum creatinine remains the AKI reference in practice. Creatinine, however, reflects kidney function and not kidney damage. This is problematic because functional changes tend to occur only after the kidney has suffered significant damage^10^. Recent studies have shown the potential of other biomarkers to be better predictors of AKI^33,46^ that are not readily measured. Indeed, it has been reported that kidney damage may begin up to 48 hours before it is detected by changes in creatinine. This fact was the motivation for the development of urine output criteria of AKI in the first place^46^.

In the realm of urine output criteria, the congruence between urine output and creatinine-based AKI is greater in MIMIC-III than in eICU. This may be a result of a much larger portion of patients that meet the creatinine-based AKI definition in MIMIC-III (54% in MIMIC-III vs 34 % in eICU). Additionally, the performance of the optimal time and volume threshold combinations both according to J-point distance and according to NRI had only a slightly better agreement with creatinine-based AKI definition than the standard urine-output based definition. We argue that the additional 4 or 6 hours of data required for this modified threshold does not merit the small improvement in classification performance.

In actuality, the relationship between urine output and creatinine is likely confounded by multiple factors. Fluctuations in urine output are also likely to be driven independently by variables completely unrelated to AKI. Overall, low urine output may translate into AKI in some patients but not in others, and potentially confounding clinical factors should be considered before urine output is used to make a diagnosis. Although it is known to be less accurate, there are known advantages to using the urine output criteria.

Ultimately AKI is a highly heterogeneous disease^29^ and it may be naïve to assume that a single feature (be it urine or creatinine) will correctly predict the same ailment for all patients. As suggested by De Corte, one future path forward may be to condition the definition of AKI on the population in question^10^. Our work presented here is a step towards incorporating this heterogeneity through physiological features.

We saw a significant improvement in the predictive performance of feature-augmented time varying GEE and GLME models with a window of 6 h (time duration of standard urine output) compared to the standard urine output based AKI definition in terms of sensitivity, specificity, and J-point distance in both databases.

Additionally, the prediction performance of all the feature-augmented time varying models consistently outperformed the prediction performance of the original urine output based definition of AKI or any refinement of its time and volume thresholds according to any of the metrics used (sensitivity, specificity, J-point distance, and NRI). Importantly, there is no trade off between any of these metrics such as an increase in specificity at the expense of sensitivity. This suggests that having a time varying model augmented with static and dynamic features is necessary for significantly improved prediction of AKI.

Furthermore, our results provide insight into features other than urine output that might improve the prediction performance of AKI. In both MIMIC and eICU, first creatinine measurement, fluid balance, and LBM were significantly associated with creatinine-based AKI. First, a higher baseline creatinine was associated with future rise in creatinine. This is a noteworthy finding as we specifically excluded patients with “abnormal” baseline creatinine—thus even a “high normal” baseline creatinine is associated with AKI. Second, positive fluid balance was associated with future rise in creatinine. It is worth noting here that we did not directly investigate the type of fluid received by the patients, which has been reported as a potential driver of AKI by others in the literature^47^. Third, a greater LBM decreased the probability of developing creatinine-based AKI. This finding is substantiated by the work of Liu et al.^48^ where they found that underweight patients had a greater chance of developing AKI in ICU as adequate nutritional intake is thought to reduce ICU length of stay and improve chances of recovery^49,50^.

In MIMIC-III, use of vasopressors and heart disease are associated with decreased risk of AKI. In MIMIC-III, more than 60% of the patients have heart disease. Of those patients, 39% were given vasopressors; Only 20% of the patients without heart disease were given vasopressors. Vasopressors stabilizes the abnormally low blood pressure and blood perfusion caused by heart disease and restores end-organ perfusion leading to better outcomes.

In eICU, use of diuretics was associated with increased chance of developing AKI. This may be due to forced diuresis leading to volume overload^51^. Also, decreased MAP had a positive association with future rise in creatinine. Low average MAP within a time window was associated with a future rise in creatinine, as expected from decreased renal perfusion. Additionally, use of vasopressors was associated with the development of AKI as also previously noted^52^. Reduction of blood flow to tissues for patients with increased fluid overload can cause harm^53^.

It is interesting to note that direction of association of a given feature depends on the underlying population. Vasopressor use was negatively associated with AKI in MIMIC whereas it was positively associated in eICU, as MIMIC has significantly more patients with heart disease diabetes, and cancer than eICU. This emphasises the importance of taking into account the patient population characteristics when making treatment decisions.

Even prior to the development of the RIFLE criteria^14^ and the AKIN modification^19^, experts remarked “none of the definitions (of AKI) used to date take into account the modifying effects of age, gender, and race on creatinine generation”^54^. Even the most recent clinical practice guidelines state that the urine output criteria are not well validated, require further investigation, and that the effects of fluid balance and other factors should be considered. One recent study of 2171 patients performs such an adjustment based on fluid balance^55^, but our work here considers fluid balance in addition to multiple other factors suggested by prior investigators.

Our findings that UO alone is not a powerful indicator of AKI but UO along with other features such as blood pressure and use of vasopressors can be a sensitive indicator are supported by Prowle et al.^56^ and Macedo et al.^57^, although it should be noted that their conclusions are based on very limited data. Prowle et al included 239 patients in their study of which 23 further developed AKI, and Macedo et al included only 75 patients of which 21 developed AKI.

Both studies sought to determine if changes in UO could be a sensitive marker of AKI using creatinine-based definition as the gold standard. However, both studies used urine output and not fluid balance to detect AKI, which is necessary as increase in fluid intake while maintaining the same UO does raise concerns about kidney function. Additionally, they used summary statistics such as mean, median and interquartile range (IQR) for continuous variables and percentages and CI for categorical variables rather than utilizing higher resolution of variables. Our modeling at a window-level rather than a patient level allows use of the appropriate corresponding values. It allows accounting for the time difference between events such as use of vasopressors and change in fluid balance as the impact of drugs lessens over time.

The last decade’s research on the topic of AKI has focused primarily on the discovery of more reliable biomarkers for laboratory diagnosis of AKI. Several biomarkers can give an indication before serum creatinine rises, but unfortunately they may perform no better than standard criteria in unselected populations, and have not been linked to improved outcomes^29,46^. Additionally, the biomarkers are not readily measured, making impossible to perform large retrospective studies on it.

With the advent of digital health records, we have the opportunity to re-calibrate consensus definitions and clinical guidelines traditionally based on expert opinion, and/or data from relatively small sample populations. This allows us to test the robustness of physiologic concepts developed based on animal experiments or studies on healthy human volunteers in the setting of critical illness. When AKIN first created a definition of AKI, large databases that relate creatinine to hourly urine output, like the Multiparameter Intelligent Monitoring in Intensive Care III database (MIMIC-III) and Collaborative Research Database (eICU), were not as readily available. Using two independent large retrospective clinical archives with significantly different patient populations we have re-examined the agreement between the two components of this definition.

While our results are robust, this improved detection cannot replace the measurement of creatinine for the definition of AKI. In the future, other definitions, and even guidelines, based on expert opinion and existing data should be revisited in this manner, based on new repositories of patient data linked with clinical outcomes, and we believe that our work presented here can serve as a prototype for this approach.

## Conclusion

In this paper, we refined the urine-based definition of AKI by optimizing urine volume and duration criteria, and also introduced a time varying detection model that incorporated physiological features that confound the relationship between hourly urine output measurements and creatinine. This was conducted using two independent data sets with different patient populations. In both data sets we consistently showed that a model which monitors repeated urine output measures in addition to other covariates (such as average MAP) has enhanced associations with future rise in creatinine, as compared to applying a fixed criterion of 0.5 ml/kg/hour of urine for 6 hours or any of its refinements. Thus, urine output and other patient characteristics could be continuously monitored in real time by a bedside algorithm. Once the multivariable definition of AKI is met in a given patient, critical steps (such as interventions to treat AKI, or adjusting the dose of medications cleared by the kidneys) could be undertaken.

## Supplementary Information

Supplementary Table S1.

## Data Availability

The openly available datasets supporting the conclusions of this article are from https://mimic.physionet.org/ and https://eicu-crd.mit.edu/.
