# Author Correction: Acute kidney injury detection using refined and physiological-feature augmented urine output

**DOI:** 10.1038/s41598-021-01415-y

**Published:** 2021-11-09

**Authors:** Sahar Alkhairy, Leo A. Celi, Mengling Feng, Andrew J. Zimolzak

**Affiliations:** 1 Massachusetts Institute of Technology, Cambridge, MA USA; 2 Beth Israel Deaconess Medical Center, Boston, MA USA; 3 Saw Swee Hock School of Public Health, National University Health System, National University of Singapore, Singapore, Singapore; 4 Baylor College of Medicine, Houston, TX USA; 5 Michael E. DeBakey VA Medical Center, Houston, TX USA

Correction to: *Scientific Reports* https://doi.org/10.1038/s41598-021-97735-0, published online 01 October 2021

The Funding section in the original version of this Article was incomplete.

“This study was supported by the National Institute of Biomedical Imaging and Bioengineering grant R01 EB001659. This study was also partially supported by the National University of Singapore Start-up Grant R-608-000-172-133.”

now reads:

“This study was supported by the National Institute of Biomedical Imaging and Bioengineering grant R01 EB001659. This study was also partially supported by the National University of Singapore Start-up Grant R-608-000-172-133; and in part by the Center for Innovations in Quality, Effectiveness and Safety (CIN 13-413), Michael E. DeBakey VA Medical Center, Houston, TX; and the Gordon and Betty Moore Foundation.”

The original Article has been corrected.

