# Supplementary material for: Acute kidney injury detection using refined and physiological-feature augmented urine output
Source: Sci Rep. 2021 Oct 1;11:19561. doi: 10.1038/s41598-021-97735-0 (PMC8486770; doi:10.1038/s41598-021-97735-0)
Supplement: Supplementary file 1 — Supplementary Table S1. [file 41598_2021_97735_MOESM1_ESM.docx]

Acute Kidney Injury Detection Using Refined and Physiological-Feature Augmented Urine Output

Sahar Alkhairy^1,^*, Leo A. Celi^1,2^, Mengling Feng^1,3, +,^ Andrew J. Zimolzak^4,5, +^

^1^ Massachusetts Institute of Technology, Cambridge, Massachusetts, USA

^2^ Beth Israel Deaconess Medical Center, Boston, Massachusetts, USA

^3^ Saw Swee Hock School of Public Health, National University Health System, National University of Singapore, Singapore

^4^ Baylor College of Medicine, Houston, Texas, USA

^5^ Michael E. DeBakey VA Medical Center, Houston, Texas, USA
^*^saharak@alum.mit.edu

^+^Joint senior authors

**Table S1:**

Combinations of time and volume thresholds were ranked based on distance from 100% sensitivity and specificity, and also ranked based on the NRI values for MIMIC-III (top) and eICU(bottom). The top combinations along with the standard pair of thresholds (0.5 ml/kg/hour for 6 hours) are listed. Combinations colored in red are based on the distance measure, and combinations colored in blue are based on NRI values. Abbreviation: NRI = net reclassification improvement.

| **MIMIC** | | | | | |
| --- | --- | --- | --- | --- | --- |
| **Time (hr)** | **Volume (ml/kg/hr)** | **Specificity** | **Sensitivity** | **Distance from 100%**  **Sensitivity and Specificity** | **NRI** |
| 12 | 0.6 | 0.578 | 0.483 | 0.668 | 0.027 |
| 10 | 0.6 | 0.5042 | 0.540 | 0.677 | 0.010 |
| 12 | 0.6 | 0.578 | 0.483 | 0.668 | 0.027 |
| 8 | 0.3 | 0.891 | 0.161 | 0.846 | 0.019 |
| 6 | 0.5 | 0.493 | 0.541 | 0.684 |  |
| **eICU** | | | | | |
| 10 | 0.6 | 0.489 | 0.484 | 0.726 | 0.026 |
| 12 | 0.7 | 0.487 | 0.473 | 0.735 | 0.014 |
| 2 | 1 | 0.074 | 0.917 | 0.929 | 0.046 |
| 2 | 0.8 | 0.097 | 0.889 | 0.910 | 0.040 |
| 6 | 0.5 | 0.382 | 0.564 | 0.756 |  |
